# 1,7-Dihydr­oxy-2,3,4-trimeth­oxy-9*H*-xanthen-9-one monohydrate from *Halenia elliptica*

**DOI:** 10.1107/S1600536808004832

**Published:** 2008-03-05

**Authors:** Peizhong Yu, Xiaojuan Shen, Changqi Hu, Edward J. Meehan, Liqing Chen

**Affiliations:** a Department of Chemistry of Natural Drugs, School of Pharmacy, Fudan University, Shanghai 200032, People’s Republic of China; b Laboratory for Structural Biology, Department of Chemistry, Graduate Programs of Biotechnology, Chemistry and Materials Science, University of Alabama, Huntsville, AL 35899, USA

## Abstract

The title compound, C_16_H_14_O_7_·H_2_O, possesses a planar three-ring skeleton; its carbonyl, one of the two hydroxy and two of the three methoxy O atoms and the water mol­ecule form hydrogen bonds, giving rise to a layer structure.

## Related literature

For the anti­depressant, anti­tumor, anti­microbial, anti­fungal, anti-inflammatory, anti­viral, cardiotonic, hypoglycemic, anti­hepatotoxic and immunomodulatory activities of simple xanthones, see: Basnet *et al.* (1994 ▶); Fernandes *et al.* (1995 ▶); Karan *et al.* (1999 ▶); Liou *et al.* (1993 ▶); Miura *et al.* (2001 ▶); Parmar *et al.* (1996 ▶); Pedro *et al.* (2002 ▶); Sousa *et al.* (2002 ▶). For the crystal structures of oxygenated xanthones, see: Evans *et al.* (2004 ▶); Gales *et al.* (2001 ▶); Jiang *et al.* (2004 ▶); Kabaleeswaran *et al.* (2003 ▶); Kato *et al.* (2005 ▶); Kijjoa *et al.* (1998 ▶); Shi *et al.* (2004 ▶, 2005 ▶); Stout *et al.* (1969 ▶); Vijayalakshmi *et al.* (1987 ▶).
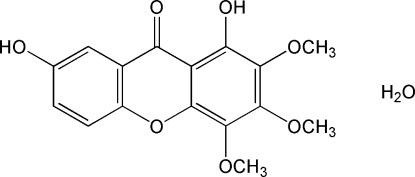


## Experimental

### 

#### Crystal data

C_16_H_14_O_7_·H_2_O
*M* *_r_* = 336.29
Monoclinic, 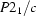

*a* = 10.9272 (9) Å
*b* = 10.4511 (8) Å
*c* = 14.0201 (11) Å
β = 111.6830 (10)°
*V* = 1487.8 (2) Å^3^
*Z* = 4
Mo *K*α radiation
μ = 0.12 mm^−1^
*T* = 298 K
0.2 × 0.1 × 0.05 mm

#### Data collection

Bruker SMART 1K CCD diffractometer
Absorption correction: none
8808 measured reflections
3563 independent reflections
2848 reflections with *I* > 2σ(*I*)
*R*_int_ = 0.052

#### Refinement

*R*[*F* ^2^ > 2σ(*F* ^2^)] = 0.046
*wR*(*F* ^2^) = 0.141
*S* = 1.06
3563 reflections
233 parameters
H atoms treated by a mixture of independent and constrained refinement
Δρ_max_ = 0.29 e Å^−3^
Δρ_min_ = −0.24 e Å^−3^

### 

Data collection: *SMART* (Bruker, 1999 ▶); cell refinement: *SAINT* (Bruker, 1999 ▶); data reduction: *SAINT*; program(s) used to solve structure: *SHELXS97* (Sheldrick, 2008 ▶); program(s) used to refine structure: *SHELXL97* (Sheldrick, 2008 ▶); molecular graphics: *SHELXTL* (Sheldrick, 2008 ▶); software used to prepare material for publication: *SHELXTL*.

## Supplementary Material

Crystal structure: contains datablocks I, global. DOI: 10.1107/S1600536808004832/ng2424sup1.cif

Structure factors: contains datablocks I. DOI: 10.1107/S1600536808004832/ng2424Isup2.hkl

Additional supplementary materials:  crystallographic information; 3D view; checkCIF report

## Figures and Tables

**Table 1:** Hydrogen-bond geometry (Å, °)

*D*—H⋯*A*	*D*—H	H⋯*A*	*D*⋯*A*	*D*—H⋯*A*
O8^i^—H16^i^⋯O2	0.73 (3)	2.58 (3)	3.091 (2)	129.4
O8^i^—H16^i^⋯O3	0.73 (3)	2.46 (3)	3.177 (2)	167.3
O8^ii^—H15^ii^⋯O6	0.84 (5)	2.08 (5)	2.923 (2)	172.3
O7—H7⋯O8^iii^	0.83	1.88	2.706 (2)	169

Symmetry codes: (i) 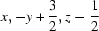; (ii) 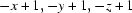; (iii) 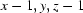.

**Fig. 1.**
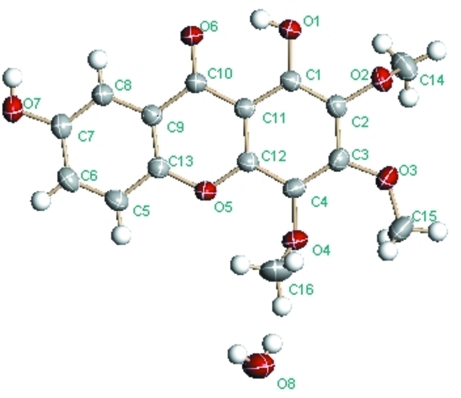
The molecular structure of (I). Displacement ellipsoids are drawn at the 50% probability level. Arbitrary atom numbering.

**Fig. 2.**
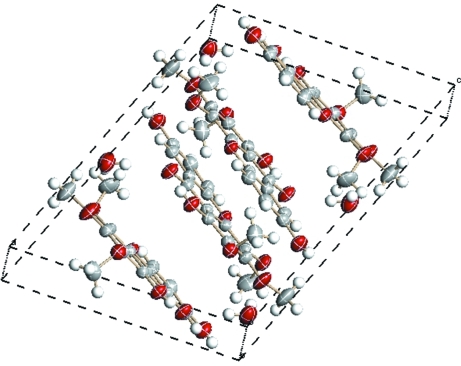
The packing of (I), viewed down the b axis.

## supplementary crystallographic information

### Comment

Xanthone compounds commonly occur in several higher plant families, such as *Gentianaceae,Guttiferae, Moraceae *and *Polygalaceae. *Simple oxygenated xanthones possess different biological activities such as antidepressant, antitumor, antimicrobial, antifungal, anti-inflammatory, antiviral, cardiotonic, hypoglycemic, antihepatotoxic and immunomodulatory (Liou *et al.*, 1993; Basnet *et al., *1994; Fernandes *et al.*, 1995; Parmar *et al.*, 1996; Karan *et al.*, 1999; Miura *et al.*, 2001; Pedro *et al., *2002; Sousa *et al., *2002). The majority of the xanthones isolated so far are hydroxyl or methoxy substituted in the xanthone skeleton. Up to present, only ten simple oxygenated xanthones were characterized by X-ray diffraction (Stout *et al., *1969; Vijayalakshmi *et al., *1987; Kijjoa *et al.*, 1998; Gales *et al., *2001; Kabaleeswaran *et al., *2003; Jiang *et al., *2004; Shi *et al., *2004; Evans *et al.*, 2004; Kato *et al.*, 2005; Shi *et al.*, 2005). 1,7-dihydroxy-2,3,4-trimethoxyxanthone (I) was first isolated from *Halenia elliptica* D. Don (Gentianaceae) and has antioxidant activity. Its crystal structure is reported for the first time in this paper. The structure of I (Figure 1) is similar to other xanthones reported with a planar three-ring skeleton. The asymmetric unit of crystal I contains one molecule I plus one water molecule. I forms hydrogen bonds with the water molecule through its carbonyl, one of the two hydroxyl and two of the three methoxyl O atoms (Table 1, Figure 2). The crystal structure is stabilized by the extensive hydrogen bond network.

### Experimental

*Halenia elliptica *D. Don was collected in *DALI*, Yunnan Province, in April 2005 and was identified by Professor Xiaokuang Ma, Department of Pharmacognosy, School of Pharmacy, *DALI* University, People's Republic of China.

Extraction and isolation: 1,7-dihydroxy-2,3,4-trimethoxyxanthone (I) was isolated from ethyl acetate fraction of the ethanol extract of the aerial parts of *Halenia elliptica* with other four 1,7-dihydroxy substituted xanthones by silica gel column chromatography with gradient mixtures of petroleum ether and ethyl acetate. Yellow crystals of I were obtained by slow evaporation of a solution in EtOH. *M*.p.164–165°C. ESI-MS *m/z* (*rel*. %): 319[*M*+H]^+.1^H-NMR (400 MHz, CDCl_3_), *d*12.60 (1*H*, *s*, OH-1), 7.60 (1*H*, *d*, *J*=3.0 Hz, H-8), 7.49 (1*H*, *d*, *J*=9.3 Hz, H-5), 7.34 (1*H*, *dd*, *J*=3.0, 9.0 Hz, H-6), 5.32 (1*H*, *br. s*, OH-7), 4.15 (3*H*, *s*, OCH_3_), 3.96 (3*H*, *s*, OCH_3_), 3.95 (3*H*, *s*, OCH_3_).

### Refinement

H atoms attached to O atoms were located in a difference map and refined with bond restraints O—H = 0.82 (2) Å. C-bound H atoms were positioned geometrically (C—H 0.93 - 0.96 Å). All H atoms were refined as riding, with *U*_iso_(H) = 1.2 - 1.5 *U*_eq_ of the parent atom.

### Crystal data

**Table:**

C_16_H_14_O_7_·H_2_O	*F*_000_ = 704
*M**_r_* = 336.29	*D*_x_ = 1.501 Mg m^−^^3^
Monoclinic, *P*2_1_/*c*	Melting point: 437 K
Hall symbol: -P 2ybc	Mo *K*α radiation λ = 0.71073 Å
*a* = 10.9272 (9) Å	Cell parameters from 3563 reflections
*b* = 10.4511 (8) Å	θ = 2.5–28.3º
*c* = 14.0201 (11) Å	µ = 0.12 mm^−^^1^
β = 111.6830 (10)º	*T* = 298 K
*V* = 1487.8 (2) Å^3^	Block, yellow
*Z* = 4	0.2 × 0.1 × 0.05 mm

### Data collection

**Table:**

Bruker SMART 1K CCD diffractometer	2848 reflections with *I* > 2σ(*I*)
Radiation source: fine-focus sealed tube	*R*_int_ = 0.053
Monochromator: graphite	θ_max_ = 28.3º
*T* = 298 K	θ_min_ = 2.5º
Thin–slice ω scans	*h* = −14→14
Absorption correction: none	*k* = −8→13
8808 measured reflections	*l* = −18→18
3563 independent reflections	

### Refinement

**Table:**

Refinement on *F*^2^	Hydrogen site location: inferred from neighbouring sites
Least-squares matrix: full	H atoms treated by a mixture of independent and constrained refinement
*R*[*F*^2^ > 2σ(*F*^2^)] = 0.046	*w* = 1/[σ^2^(*F*_o_^2^) + (0.0743*P*)^2^ + 0.2855*P*] where *P* = (*F*_o_^2^ + 2*F*_c_^2^)/3
*wR*(*F*^2^) = 0.142	(Δ/σ)_max_ < 0.001
*S* = 1.06	Δρ_max_ = 0.29 e Å^−^^3^
3563 reflections	Δρ_min_ = −0.24 e Å^−^^3^
233 parameters	Extinction correction: SHELXL97 (Sheldrick, 2008), Fc^*^=kFc[1+0.001xFc^2^λ^3^/sin(2θ)]^-1/4^
Primary atom site location: structure-invariant direct methods	Extinction coefficient: 0.0085 (18)
Secondary atom site location: difference Fourier map	

### Special details

**Table:**

Geometry. All e.s.d.'s (except the e.s.d. in the dihedral angle between two l.s. planes) are estimated using the full covariance matrix. The cell e.s.d.'s are taken into account individually in the estimation of e.s.d.'s in distances, angles and torsion angles; correlations between e.s.d.'s in cell parameters are only used when they are defined by crystal symmetry. An approximate (isotropic) treatment of cell e.s.d.'s is used for estimating e.s.d.'s involving l.s. planes.
Refinement. Refinement of *F*^2^ against ALL reflections. The weighted *R*-factor *wR* and goodness of fit *S* are based on *F*^2^, conventional *R*-factors *R* are based on *F*, with *F* set to zero for negative *F*^2^. The threshold expression of *F*^2^ > σ(*F*^2^) is used only for calculating *R*-factors(gt) *etc*. and is not relevant to the choice of reflections for refinement. *R*-factors based on *F*^2^ are statistically about twice as large as those based on *F*, and *R*- factors based on ALL data will be even larger.

### Fractional atomic coordinates and isotropic or equivalent isotropic displacement parameters (Å^2^)

**Table:**

	*x*	*y*	*z*	*U*_iso_*/*U*_eq_	
O5	0.62447 (9)	0.47253 (9)	0.17678 (8)	0.0374 (2)	
O1	0.44791 (10)	0.90069 (10)	0.12081 (9)	0.0441 (3)	
H1	0.3758	0.8590	0.0943	0.066*	
C11	0.52749 (12)	0.68367 (13)	0.14635 (9)	0.0315 (3)	
O6	0.29738 (10)	0.70239 (10)	0.06007 (9)	0.0492 (3)	
O2	0.69716 (11)	0.99477 (10)	0.21570 (8)	0.0437 (3)	
C2	0.67515 (14)	0.86433 (13)	0.20640 (10)	0.0361 (3)	
C12	0.63679 (13)	0.60211 (13)	0.18592 (10)	0.0325 (3)	
C10	0.39784 (13)	0.63185 (13)	0.09340 (10)	0.0343 (3)	
C13	0.50227 (13)	0.41989 (13)	0.12579 (10)	0.0336 (3)	
C4	0.76454 (13)	0.64776 (14)	0.23766 (11)	0.0370 (3)	
C8	0.26918 (14)	0.43164 (14)	0.02687 (11)	0.0371 (3)	
H8	0.1934	0.4798	−0.0049	0.045*	
O3	0.89800 (11)	0.84290 (12)	0.29620 (11)	0.0599 (4)	
C3	0.78279 (13)	0.78037 (15)	0.24786 (11)	0.0385 (3)	
C9	0.38925 (13)	0.49338 (13)	0.08075 (10)	0.0331 (3)	
C1	0.54949 (13)	0.81769 (13)	0.15801 (10)	0.0331 (3)	
O7	0.14973 (11)	0.23466 (11)	−0.03034 (10)	0.0517 (3)	
H7	0.0882	0.2864	−0.0560	0.078*	
C7	0.26315 (14)	0.30003 (14)	0.02086 (11)	0.0386 (3)	
O4	0.86845 (10)	0.56302 (11)	0.26990 (9)	0.0500 (3)	
C5	0.49582 (15)	0.28688 (14)	0.12202 (11)	0.0398 (3)	
H5	0.5711	0.2383	0.1543	0.048*	
C6	0.37723 (16)	0.22793 (14)	0.07010 (11)	0.0421 (3)	
H6	0.3727	0.1391	0.0677	0.051*	
C15	1.02209 (16)	0.7842 (2)	0.32996 (18)	0.0673 (5)	
H15A	1.0321	0.7309	0.3881	0.101*	
H15B	1.0895	0.8486	0.3492	0.101*	
H15C	1.0297	0.7329	0.2756	0.101*	
C14	0.7299 (2)	1.04398 (18)	0.13328 (16)	0.0595 (5)	
H14A	0.8034	0.9974	0.1290	0.089*	
H14B	0.7526	1.1328	0.1453	0.089*	
H14C	0.6557	1.0349	0.0700	0.089*	
C16	0.8790 (2)	0.4892 (2)	0.35909 (16)	0.0701 (6)	
H16A	0.8795	0.5457	0.4132	0.105*	
H16B	0.9592	0.4406	0.3811	0.105*	
H16C	0.8052	0.4321	0.3425	0.105*	
O8	0.92795 (15)	0.37653 (18)	0.88918 (14)	0.0695 (4)	
H15	0.862 (4)	0.361 (4)	0.905 (3)	0.123 (12)*	
H16	0.912 (3)	0.436 (3)	0.860 (2)	0.104 (12)*	

### Atomic displacement parameters (Å^2^)

**Table:**

	*U*^11^	*U*^22^	*U*^33^	*U*^12^	*U*^13^	*U*^23^
O5	0.0319 (5)	0.0313 (5)	0.0448 (5)	0.0041 (4)	0.0093 (4)	0.0004 (4)
O1	0.0363 (5)	0.0311 (5)	0.0567 (6)	0.0041 (4)	0.0076 (5)	0.0031 (4)
C11	0.0310 (6)	0.0309 (6)	0.0311 (6)	0.0016 (5)	0.0098 (5)	0.0008 (5)
O6	0.0318 (5)	0.0347 (5)	0.0694 (7)	0.0041 (4)	0.0048 (5)	0.0046 (5)
O2	0.0460 (6)	0.0323 (5)	0.0512 (6)	−0.0056 (4)	0.0160 (5)	−0.0051 (4)
C2	0.0382 (7)	0.0318 (6)	0.0374 (7)	−0.0016 (5)	0.0128 (6)	−0.0024 (5)
C12	0.0329 (6)	0.0314 (6)	0.0328 (6)	0.0024 (5)	0.0116 (5)	−0.0001 (5)
C10	0.0322 (6)	0.0318 (6)	0.0359 (6)	0.0017 (5)	0.0089 (5)	0.0031 (5)
C13	0.0348 (7)	0.0328 (7)	0.0336 (6)	0.0019 (5)	0.0132 (5)	−0.0006 (5)
C4	0.0297 (6)	0.0379 (7)	0.0396 (7)	0.0051 (5)	0.0086 (5)	0.0001 (5)
C8	0.0352 (7)	0.0341 (7)	0.0393 (7)	−0.0005 (5)	0.0107 (6)	0.0001 (5)
O3	0.0317 (5)	0.0457 (7)	0.0854 (9)	−0.0031 (5)	0.0018 (5)	−0.0102 (6)
C3	0.0318 (6)	0.0401 (7)	0.0401 (7)	−0.0028 (5)	0.0091 (5)	−0.0042 (6)
C9	0.0345 (7)	0.0314 (6)	0.0328 (6)	0.0006 (5)	0.0119 (5)	0.0011 (5)
C1	0.0342 (6)	0.0310 (6)	0.0333 (6)	0.0024 (5)	0.0113 (5)	0.0014 (5)
O7	0.0443 (6)	0.0396 (6)	0.0659 (7)	−0.0102 (5)	0.0139 (5)	−0.0092 (5)
C7	0.0422 (7)	0.0356 (7)	0.0398 (7)	−0.0061 (6)	0.0175 (6)	−0.0047 (6)
O4	0.0329 (5)	0.0435 (6)	0.0661 (7)	0.0095 (4)	0.0095 (5)	0.0028 (5)
C5	0.0433 (7)	0.0315 (7)	0.0449 (7)	0.0061 (6)	0.0166 (6)	0.0003 (6)
C6	0.0519 (8)	0.0294 (6)	0.0483 (8)	−0.0010 (6)	0.0222 (7)	−0.0033 (6)
C15	0.0329 (8)	0.0638 (12)	0.0911 (14)	−0.0019 (8)	0.0063 (8)	−0.0113 (10)
C14	0.0677 (11)	0.0416 (9)	0.0790 (12)	−0.0009 (8)	0.0387 (10)	0.0066 (8)
C16	0.0575 (11)	0.0644 (12)	0.0714 (12)	0.0185 (9)	0.0038 (9)	0.0198 (10)
O8	0.0527 (8)	0.0662 (10)	0.0874 (11)	0.0122 (7)	0.0232 (7)	0.0162 (8)

### Geometric parameters (Å, °)

**Table:**

O5—C12	1.3623 (16)	O3—C3	1.3567 (17)
O5—C13	1.3753 (17)	O3—C15	1.402 (2)
O1—C1	1.3523 (16)	O7—C7	1.3640 (17)
O1—H1	0.8561	O7—H7	0.8336
C11—C12	1.4040 (18)	C7—C6	1.401 (2)
C11—C1	1.4204 (19)	O4—C16	1.437 (2)
C11—C10	1.4402 (18)	C5—C6	1.375 (2)
O6—C10	1.2601 (16)	C5—H5	0.9300
O2—C2	1.3820 (17)	C6—H6	0.9300
O2—C14	1.426 (2)	C15—H15A	0.9600
C2—C1	1.3765 (19)	C15—H15B	0.9600
C2—C3	1.409 (2)	C15—H15C	0.9600
C12—C4	1.3979 (19)	C14—H14A	0.9600
C10—C9	1.4568 (19)	C14—H14B	0.9600
C13—C5	1.3919 (19)	C14—H14C	0.9600
C13—C9	1.3920 (19)	C16—H16A	0.9600
C4—O4	1.3779 (16)	C16—H16B	0.9600
C4—C3	1.400 (2)	C16—H16C	0.9600
C8—C7	1.3781 (19)	O8—H15	0.85 (4)
C8—C9	1.4057 (19)	O8—H16	0.73 (4)
C8—H8	0.9300		
			
C12—O5—C13	119.33 (10)	O1—C1—C11	120.54 (12)
C1—O1—H1	109.5	C2—C1—C11	120.10 (12)
C12—C11—C1	118.04 (12)	C7—O7—H7	109.5
C12—C11—C10	120.44 (12)	O7—C7—C8	123.07 (14)
C1—C11—C10	121.51 (12)	O7—C7—C6	117.39 (13)
C2—O2—C14	111.49 (12)	C8—C7—C6	119.54 (13)
C1—C2—O2	120.20 (12)	C4—O4—C16	114.94 (13)
C1—C2—C3	120.75 (13)	C6—C5—C13	119.48 (14)
O2—C2—C3	119.05 (12)	C6—C5—H5	120.3
O5—C12—C4	115.67 (12)	C13—C5—H5	120.3
O5—C12—C11	121.75 (12)	C5—C6—C7	120.84 (13)
C4—C12—C11	122.58 (13)	C5—C6—H6	119.6
O6—C10—C11	121.83 (12)	C7—C6—H6	119.6
O6—C10—C9	121.87 (12)	O3—C15—H15A	109.5
C11—C10—C9	116.31 (12)	O3—C15—H15B	109.5
O5—C13—C5	116.43 (12)	H15A—C15—H15B	109.5
O5—C13—C9	122.92 (12)	O3—C15—H15C	109.5
C5—C13—C9	120.65 (13)	H15A—C15—H15C	109.5
O4—C4—C12	119.65 (13)	H15B—C15—H15C	109.5
O4—C4—C3	122.26 (12)	O2—C14—H14A	109.5
C12—C4—C3	117.91 (12)	O2—C14—H14B	109.5
C7—C8—C9	120.33 (13)	H14A—C14—H14B	109.5
C7—C8—H8	119.8	O2—C14—H14C	109.5
C9—C8—H8	119.8	H14A—C14—H14C	109.5
C3—O3—C15	124.13 (14)	H14B—C14—H14C	109.5
O3—C3—C4	126.78 (13)	O4—C16—H16A	109.5
O3—C3—C2	112.65 (13)	O4—C16—H16B	109.5
C4—C3—C2	120.57 (12)	H16A—C16—H16B	109.5
C13—C9—C8	119.08 (12)	O4—C16—H16C	109.5
C13—C9—C10	119.11 (12)	H16A—C16—H16C	109.5
C8—C9—C10	121.79 (12)	H16B—C16—H16C	109.5
O1—C1—C2	119.35 (12)	H15—O8—H16	106 (3)
			
C14—O2—C2—C1	−93.37 (16)	O5—C13—C9—C8	177.67 (12)
C14—O2—C2—C3	87.18 (17)	C5—C13—C9—C8	−3.1 (2)
C13—O5—C12—C4	−179.21 (11)	O5—C13—C9—C10	−4.2 (2)
C13—O5—C12—C11	1.25 (19)	C5—C13—C9—C10	175.03 (12)
C1—C11—C12—O5	−179.12 (11)	C7—C8—C9—C13	1.5 (2)
C10—C11—C12—O5	−0.6 (2)	C7—C8—C9—C10	−176.60 (13)
C1—C11—C12—C4	1.4 (2)	O6—C10—C9—C13	−175.33 (13)
C10—C11—C12—C4	179.86 (12)	C11—C10—C9—C13	4.52 (19)
C12—C11—C10—O6	177.61 (13)	O6—C10—C9—C8	2.7 (2)
C1—C11—C10—O6	−4.0 (2)	C11—C10—C9—C8	−177.40 (12)
C12—C11—C10—C9	−2.24 (18)	O2—C2—C1—O1	−0.8 (2)
C1—C11—C10—C9	176.19 (12)	C3—C2—C1—O1	178.70 (12)
C12—O5—C13—C5	−178.02 (12)	O2—C2—C1—C11	178.48 (12)
C12—O5—C13—C9	1.24 (19)	C3—C2—C1—C11	−2.1 (2)
O5—C12—C4—O4	3.85 (19)	C12—C11—C1—O1	179.59 (11)
C11—C12—C4—O4	−176.61 (12)	C10—C11—C1—O1	1.1 (2)
O5—C12—C4—C3	179.11 (12)	C12—C11—C1—C2	0.37 (19)
C11—C12—C4—C3	−1.4 (2)	C10—C11—C1—C2	−178.10 (12)
C15—O3—C3—C4	10.8 (3)	C9—C8—C7—O7	−179.92 (13)
C15—O3—C3—C2	−169.68 (17)	C9—C8—C7—C6	0.9 (2)
O4—C4—C3—O3	−5.7 (2)	C12—C4—O4—C16	−75.54 (18)
C12—C4—C3—O3	179.12 (14)	C3—C4—O4—C16	109.41 (18)
O4—C4—C3—C2	174.75 (13)	O5—C13—C5—C6	−178.43 (12)
C12—C4—C3—C2	−0.4 (2)	C9—C13—C5—C6	2.3 (2)
C1—C2—C3—O3	−177.47 (13)	C13—C5—C6—C7	0.2 (2)
O2—C2—C3—O3	1.98 (19)	O7—C7—C6—C5	179.03 (13)
C1—C2—C3—C4	2.1 (2)	C8—C7—C6—C5	−1.8 (2)
O2—C2—C3—C4	−178.45 (12)		

### Hydrogen-bond geometry (Å, °)

**Table:**

*D*—H···*A*	*D*—H	H···*A*	*D*···*A*	*D*—H···*A*
O8^i^—H16^i^···O2	0.73 (3)	2.58 (3)	3.091 (2)	129.4
O8^i^—H16^i^···O3	0.73 (3)	2.46 (3)	3.177 (2)	167.3
O8^ii^—H15^ii^···O6	0.84 (5)	2.08 (5)	2.923 (2)	172.3
O7—H7···O8^iii^	0.83	1.88	2.706 (2)	169

Symmetry codes: (i) *x*, −*y*+3/2, *z*−1/2; (ii) −*x*+1, −*y*+1, −*z*+1; (iii) *x*−1, *y*, *z*−1.
